# Comparative kinetic analysis of two fungal β-glucosidases

**DOI:** 10.1186/1754-6834-3-3

**Published:** 2010-02-11

**Authors:** Marie Chauve, Hugues Mathis, Delphine Huc, Dominique Casanave, Frédéric Monot, Nicolas Lopes Ferreira

**Affiliations:** 1 IFP, Biotechnology Department, Avenue de Bois-Préau 92852 Rueil-Malmaison, France; 2 IFP, Reaction and Reactor Modeling Department, Rond Point de l'Echangeur 69360 Solaize, France

## Abstract

**Background:**

The enzymatic hydrolysis of cellulose is still considered as one of the main limiting steps of the biological production of biofuels from lignocellulosic biomass. It is a complex multistep process, and various kinetic models have been proposed. The cellulase enzymatic cocktail secreted by *Trichoderma reesei *has been intensively investigated. β-glucosidases are one of a number of cellulolytic enzymes, and catalyze the last step releasing glucose from the inhibitory cellobiose. β-glucosidase (BGL1) is very poorly secreted by *Trichoderma reesei *strains, and complete hydrolysis of cellulose often requires supplementation with a commercial β-glucosidase preparation such as that from *Aspergillus niger *(Novozymes SP188). Surprisingly, kinetic modeling of β-glucosidases lacks reliable data, and the possible differences between native *T. reesei *and supplemented β-glucosidases are not taken into consideration, possibly because of the difficulty of purifying BGL1.

**Results:**

A comparative kinetic analysis of β-glucosidase from *Aspergillus niger *and BGL1 from *Trichoderma reesei*, purified using a new and efficient fast protein liquid chromatography protocol, was performed. This purification is characterized by two major steps, including the adsorption of the major cellulases onto crystalline cellulose, and a final purification factor of 53. Quantitative analysis of the resulting β-glucosidase fraction from *T. reesei *showed it to be 95% pure. Kinetic parameters were determined using cellobiose and a chromogenic artificial substrate. A new method allowing easy and rapid determination of the kinetic parameters was also developed. β-Glucosidase SP188 (K_m _= 0.57 mM; K_p _= 2.70 mM) has a lower specific activity than BGL1 (K_m _= 0.38 mM; K_p _= 3.25 mM) and is also more sensitive to glucose inhibition. A Michaelis-Menten model integrating competitive inhibition by the product (glucose) has been validated and is able to predict the β-glucosidase activity of both enzymes.

**Conclusions:**

This article provides a useful comparison between the activity of β-glucosidases from two different fungi, and shows the importance of fully characterizing both enzymes. A Michaelis-Menten model was developed, including glucose inhibition and kinetic parameters, which were accurately determined and compared. This model can be further integrated into a cellulose hydrolysis model dissociating β-glucosidase activity from that of other cellulases. It can also help to define the optimal enzymatic cocktails for new β-glucosidase activities.

## Introduction

Discovered four decades ago by Reese and Mandels [1,2], the cellulolytic enzyme system secreted by the filamentous fungi *Trichoderma reesei *(synonym *Trichoderma viride*) is the initial parent of most fungal strains for industrial cellulase production. The hydrolysis step converting cellulose to glucose is recognized as the major limiting step in the development of biological processes for production of biofuels from lignocellulosic raw materials because of the low efficiency of cellulases and their cost.

The enzymatic hydrolysis of cellulose involves three types of cellulases (cellobiohydrolases, endoglucanases and β-glucosidases) working in synergy [3]. Endoglucanases (EC 3.2.1.4) randomly cleave the β-1,4 glycosidic linkages of cellulose; cellobiohydrolases (EC 3.2.1.91) attack cellulose chain ends to produce the constitutive unit of cellulose, cellobiose (a dimer of glucose linked by a β-1,4 glycosidic bond); and β-glucosidases (EC 3.2.1 21) hydrolyse cellobiose into two molecules of glucose. In fact, enzymatic hydrolysis of cellulose is a six-step complex process, the last step being a homogenous catalysis reaction involving the action of β-glucosidase on cellobiose. Cellobiose is a strong inhibitor of both cellobiohydrolases and endocellulases, and the β-glucosidase action can reduce its effect. In addition, the produced glucose also inhibits cellulolysis, although to a lesser extent [4]. Unexpectedly, the amount of β-glucosidase-1 (BGL1) generated by *T. reesei *hyperproducing strains represents a very low percentage of the total secreted proteins [3,5]. This very low level of β-glucosidase activity [6] often limits the amount of this enzyme in commercial cellulase preparations. This limitation can be alleviated either by overexpressing β-glucosidase in *T. reesei *or by adding extra β-glucosidase from other sources [7,8]. Supplementing the native *T. reesei *enzymatic cocktail with β-glucosidase from other fungi is often performed to avoid inhibition of cellobiose in standardized hydrolysis test [9]. The most common commercial β-glucosidase preparation (Novozymes SP188; Novo Nodisk A/S, Bagsvaerd, Danmark) is produced by *Aspergillus niger*.

Modeling enzymatic hydrolysis kinetics is necessary to better characterize and understand the interactions between cellulases and cellulose, to integrate present and future enzyme improvements, and to design optimized process reactors and equipments. Surprisingly, although kinetic modeling of cellulose enzymatic hydrolysis is a subject of intensive studies, as reviewed by Zhang and Lynd [10] in 2004 and more recently by Bansal *et al*. [11], kinetic data on β-glucosidase are difficult to exploit. Kinetic parameters, including inhibition by cellobiose, determined using the Michaelis-Menten (M-M) model, are presented in Table 1. Their limitations include lack of homogeneity in the methods used and in the origin of the enzyme. The kinetics of commercial β-glucosidase from *A. niger *(mainly Novozymes SP188) have been more fully investigated with respect to the β-glucosidase from *T. reesei*. The choice of crude samples or highly purified enzymes or of natural or synthetic substrates, and the introduction of the product inhibition parameter strongly influence the obtained values.

**Table 1:** Kinetic parameters of β-glucosidase from *Aspergillus niger *and *Trichoderma reesei*

Enzyme	T,°C	Ea, kJ/mol	Substrate	Inhibition	K_m_, mM	Kp,mM	K_m_/Kp	Ref
Novozymes S188 (A. niger)	50	-	pNPG	-	0.46	-	-	[17]
					0.36	-	-	

Sigma (*A. niger*)	25	-	pNPG	C	1	3	0.33	[19]
			Cellobiose		2.7	-	-	

Novozymes S188	50	52.5	pNPG	C	1.03	3	0.34	[4]
	20 to 80		Cellobiose		5.63	-	-	

*A. niger *(Culture)	30	-	pNPG	C	0.64	3.4	0.19	[20]

Cellobiase 250L (A. niger)	50	-	Cellobiose	C + S	1.66	2.87	0.57	[12]
*T. reesei *Rut C30					0.153	0.212	0.72	

Novozymes S188	50	46	Cellobiose	C - UC - NC + S	-	-	-	[21]

*T. reesei *(Culture)	50	-	pNPG	C	0.182	0.624	0.29	[22]
			Cellobiose		2.1	-	-	

*T. reesei *QM9414	40	-	pNPG	C	0.102	0.7	0.14	[23]
			Cellobiose		1.25	-	-	

Ea, Activation energy; C, competitive; NC, non-competitive; S, substrate inhibition; UC, uncompetitive

At this stage, a comparison of β-glucosidases from *A. niger *and *T. reesei *is required to validate a model of cellulose hydrolysis using cellulase mixtures supplemented or not with β-glucosidase. To our knowledge, both enzymes have been previously compared only once [12], and without any previous purification, which can greatly affect the values of the kinetic constants determined and the representativeness of the deduced models. We focused our study on the parameters important for future industrial utilization of the enzymes in a biofuel production process, especially in quantifying the effects of cellobiose, glucose and temperature. Because β-glucosidase from *T. reesei *represents a very low proportion of the cellulolytic mixture secreted, a procedure for its purification was developed. In addition, a specific method suitable for a rapid reaction time course was also used. This led to the first consistent comparative kinetic analysis between *A. niger *and *T. reesei *β-glucosidase on their natural and artificial substrates, and can be further used for modeling addition of extra β-glucosidase.

## Methods

### Enzyme samples, substrates, buffer and reagents

A commercial β-glucosidase preparation (SP188; Novozymes), microcrystalline cellulose (Avicel PH101), cellobiose, p-nitrophenol and ρ-nitrophenyl-β-D-glucopyranoside (pNPG) used in this study were purchased from Sigma-Aldrich (Sigma-Aldrich, Lyon, France). Glucose was obtained from Prolabo. All buffer components and salts used were reagent grade and obtained from Sigma Aldrich or GE Healthcare (GE Healthcare, Saclay, France) for enzyme purification.

*T. reesei *β-glucosidase was obtained from a crude enzyme preparation using the hypercellulolytic mutant strain CL847 [13]. Enzymatic productions were performed in 2.5 L working volume fermentors using two steps: (i) growth on glucose,(ii) fed batch with a sugar mixture containing glucose, lactose and xylose as the carbon source, as previously described [5,14]. At the end of the fermentation, the mycelia were removed by centrifugation from the culture medium at 20,000 g and the clear medium containing the enzymes secreted by *T. reesei *(40 mg proteins per mL) was further used for purification of β-glucosidase.

### Preparation of β-glucosidase from SP188 and *T. reesei CL847*

Fast protein liquid chromatography (Akta FPLC System; GE Healthcare) was used for the following purification experiments.

For biochemical characterization of the β-glucosidase, the SP188 commercial preparation was desalted to remove the ingredients present in the industrial formulation. For this purification step, a desalting column (Hi-trap; GE Healthcare) previously equilibrated with 25 mM imidazole pH 7.6 was used.

The purified *T. reesei *β-glucosidase used in this study was prepared as follows. A sample (0.625 mL) of the cell-free medium containing 25 mg of extracellular proteins was mixed for 30 minutes at 4°C with 20 mL of 50 mM citrate buffer pH 4.8 containing 5% Avicel PH101. After centrifugation (6000 g for 10 minutes at 4°C), the supernatant was recovered; this step was repeated once using the same amount of fresh Avicel. This allows the adsorption of proteins containing a cellulose binding domain (> 80% of the total protein content of the initial crude sample). The resulting supernatant was concentrated using a centrifuge (Vivaspin 20; Sartorius stedim, Aubagne, France) with a molecular weight cut-off of 50 kDa, allowing separation between β-glucosidase and any remaining low molecular weight proteins. This β-glucosidase-enriched sample was then desalted as described for SP188. The desalted sample was then injected into a column (10/100 GL; MonoQ) previously equilibrated with 25 mM imidazole pH 7.6 (linear flow rate of 0.7 mL/min) using FPLC. Because β-glucosidase has a high apparent isoelectric point (pI) value (close to 8), highly purified β-glucosidase was recovered by collecting the non-retained fraction. The pH of the β-glucosidase fraction was adjusted to pH 4.8 using 1 M HCl for storage. When necessary, qualitative analysis of the sample was performed using one-dimensional (1D) gel electrophoresis according to Laemmli [15], and protein concentration was measured according to Lowry [16], using bovine serum albumin as the protein standard.

### Enzyme assays

β-glucosidase activity was determined by measuring the amount of glucose and *p*-nitrophenol released, respectively, from cellobiose and *p*-nitrophenyl-β-D-glucopyranoside (pNPG) used as substrates. Native substrate assays (duplicate) involved incubating the substrate at concentrations from 0.23 to 16.4 mM in 50 mM citrate buffer pH 4.8 and enzyme solution (in a range from 2.5 to 10 mg/L) at 50°C with a time interval of 5 minutes and a total period of 20 minutes. The final volume of the assays was 100 μL and they were carried out in 1.5 mL Eppendorf tubes. The reaction was stopped by heating at 95°C for 5 min and cooled to 4°C. pNPG triplicate assays were performed using the same protocol as above but using a substrate range from 0.15 to 5 mM and and enzyme concentration of 1.25 to 5 mg/L. Reactions were prepared in 96-well PCR plates, and incubations were performed in a thermal cycler (PCR i-Cycler; Bio-Rad Laboratories, Marnes la coquette, France). Enzymatic activity was stopped by adding 100 μL of a 2% Na_2_CO_3 _solution.

For both pNPG and cellobiose substrates, inhibition parameters were determined using the same concentrations as above and glucose concentration up to 1 g/L. Inhibition effect of higher glucose concentrations (up to 60 g/L), was tested with pNPG as substrate.

The thermal stability was investigated by heating the enzyme to 50°C, removing aliquots every 24 h for 72 h in total, and assaying for kinetic measurement with 5 mM pNPG. Results showed no loss of activity within 72 h for both enzymes at 50°C (data not shown). Six additional experiments, in the validation domain of the model, were performed to evaluate the quality of the model prediction. These assays were not used to fit the parameters of the model.

### Analytical methods

Glucose concentration was determined using a high performance liquid chromatography system (ISC300; Dionex, Jouy En Josas, France) equipped with an amperometric detector. Data acquisition from the detector and determination of retention times and peak areas were performed using Chromeleon software (Dionex). A chromatography column (CarboPac PA1; measuring 250 mm × 4 mm internal diameter) with polystyrene-divinylbenzenzene pellicular resin (particle size 10 μm) and a pressure limit of 2000 psi was used. Purified water (18 MΩ; Milli-Q Plus Water Purification System; Millipore, Bedford, MA, USA) was used to prepare all solutions. Eluent 1 was 200 mM NaOH (Fischer Scientific, Illkirch, France) and eluent 2 was water (flow-rate: 1.0 mL/min). The detector operated in the amperometric mode using a gold working electrode and an Ag/AgCl pH reference electrode. Glucose, xylose and cellobiose solutions at 10 mg/L were used as external standards.

*p*-Nitrophenol was quantified by spectrophotometry at 405 nm (Multiskan Ascent Scanner; Thermo Labsystem, Issy les moulineaux, France) using 96-well plates. A range of *p*-nitrophenol solutions from 0 to 0.3 mM was used for calibration.

Km values were determined from the mean value of rates (in duplicates or triplicates) at six concentrations of substrate (PNPG or cellobiose), and Ki values were determined by the same procedure with four concentrations of glucose.

## Results and discussion

### Purification of β-glucosidases

**Figure 1:**
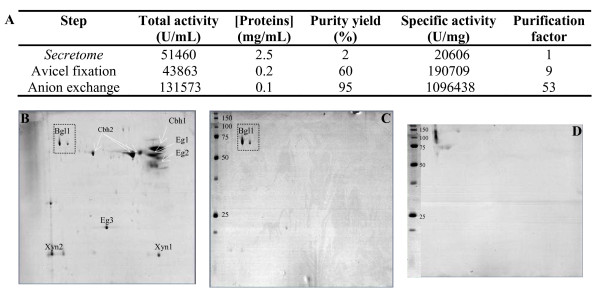
**Purification of β-glucosidase from *T. reesei***. (a) Purification procedure; mean values of duplicates are presented. Relative standard deviation was < 5% in all cases. 2D gel electrophoresis of (b) supernatant from *T. reesei*, (c) purified *T. reesei *β-glucosidase; (d) SP188. Molecular weight markers are given in kDa.

The purity of the desalted SP188 sample was checked using two-dimensional (2D) gel electrophoresis (Figure 1). We were able to distinguish two major bands, corresponding to the isoforms of the β-glucosidases previously described [17], and three minor bands (estimated purity yield close to 85% of the total protein content; data not shown).

Purification of *T. reesei *β-glucosidase may appear challenging because it represents < 2% of the secretome of hyperproducing strains [5,17], which mainly contains cellobiohydrolases and endoglucanases. The whole purification procedure is detailed in Figure 1. The method we used does not involve any fixation of the target enzyme onto a column, so that it preserves its biochemical integrity. It takes advantage of the capacity of processive and non-processive cellulases to adsorb onto cellulosic polymers via their cellulose binding domains [18], which are absent in *T. reesei *BGL1. With this first purification step, 80% of the proteins contained in the original crude were trapped. Taking into account the theoretical and apparent molecular weight of β-glucosidase (close to 75 and 117 kDa, respectively), the enriched sample was concentrated using a 50 kDa cut-off membrane. Qualitative analysis was performed using 1D gel electrophoresis of the recovered fraction. We were able to detect a clear major band corresponding to the β-glucosidase and two other remaining proteins (estimated purity yield close to 60%) with apparent molecular weights of 100 kDa and 20 kDa. Using the pI of β-glucosidase, the concentrated sample was finally purified using a monoQ anionic column equilibrated at pH 7.4, to trap the last contaminating proteins in the sample. As described by Herpoël-Gimbert *et al*. [5], quantitative analysis of the resulting β-glucosidase fraction revealed a degree of purity of 95%.

### Full kinetic model using β-glucosidase from *A. niger*

**Figure 2:**
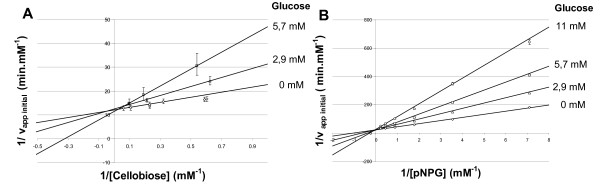
**Lineweaver-Burk plots**. (a) Cellobiose and (b) pNPG with *A. niger *β-glucosidase.

Synthetic (pNPG) and natural (cellobiose) substrates were used for the kinetic model. The reaction rate of the commercial enzyme for both substrates, calculated from the slopes of glucose vs. time data curves, are presented in a Lineweaver-Burk plot (Figure 2). The classic common intercept on the *y *axis for different glucose confirms their competitive inhibition by glucose, involving the following mechanism with two reactions described in equations 1 and 2,(1)(2)

where E is the enzyme, S is the substrate, ES is the enzyme-substrate complex, and P is the product. In the first step of the mechanism, β-glucosidase and the substrate form a complex allowing the β(1,4) glucosidic bond cleavage. Competitive inhibition involves the formation of an inactive enzyme-product complex and this seems to be independent of the substrate nature. Under the pseudosteady-state expression, the expression of the initial rate is given by equation 3,(3)

where [S]^0 ^is the initial substrate concentration (mM), [P]^0 ^is the initial product concentration (mM), [E]^0 ^is the initial enzyme concentration (mM, with M_β-glucosidase _= 117 kDa [17]), K_m _is the M-M constant (mM), K_p _is the inhibition constant (mM), k_cat _is the first order kinetic constant (per minute), and r_ini _is the initial rate of hydrolysis (mM/min)

The characteristics of the *A. niger *β-glucosidase obtained by numerical integration of equation (3) using the least-squares method, are summarized in Table 2. First kinetic parameters for both substrates were compared to validate the use of pNPG as substrate. *K*_*m *_and ratio *K*_*m*_/*K*_*p *_values found for both substrates are close to literature values (Table 2) and differences can be explained by the purification method used, which maintains the integrity of the enzyme. The lower apparent M-M constant of β-glucosidase for pNPG showed that the enzyme had a higher affinity for the synthetic substrate. The *K*_*m*_/*K*_*p *_ratio was then calculated to identify the inhibitor influence, and this revealed a strong inhibition of glucose using both substrates. According to the inhibition mechanism, the formation of an inactive enzyme-product complex does not seem to be dependent on the substrate nature. The preservation of the *K*_*m*_/*K*_*p *_ratio clearly demonstrates the necessity to use a model integrating a competitive inhibition mechanism. pNPG was then used to study β-glucosidase activity, and a PCR plate method was developed to simplify the experimentation procedure.

**Table 2:** Kinetic parameters of the *A. niger *β-glucosidase preparation using a Michaelis-Menten model

Property	Cellobiose	pNPG
*K*_*m*_, mM	0.88	0.57
*K*_*p *_competitive inhibition, mM	3.40	2.70
*K*_*m*_/*K*_*p*_	0.26	0.21

To have a full kinetic model suitable for hydrolysis process design, an important parameter is the effect of the temperature on the activity of the enzymes. Hydrolysis of cellobiose was performed at temperatures from 30 to 70°C to determine the pre-exponential factor and the activation energy in Arrhenius equation (4),(4)

where *k*_*cat*_: first order kinetic constant (per min), *A *is the pre-exponential factor, *E*_*a *_is the activation energy (J/mol), *R *is the universal gas constant (8.314 J/mol/K), and *T *is the temperature (°K)

**Figure 3:**
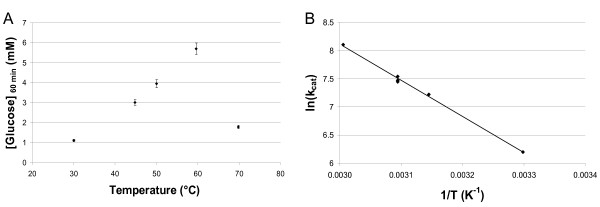
**Influence of temperature on the enzymatic activity of the *A. niger *β-glucosidase on cellobiose**. (a) Glucose concentration produced in 60 minutes as a function of temperature for β-glucosidase at the concentration of 5.1 mg/L in 16 mM cellobiose. (b) Arrhenius plot of *A. niger *β-glucosidase at a range of temperatures between 30 and 60°C (*r *= 0.9955).

Figure 3 shows the glucose concentration produced by the *A. niger *β-glucosidase as a function of temperature, and the Arrhenius plot for this enzyme. The optimum enzyme activity was observed at around 65°C using cellobiose as a substrate, which is in agreement with Dekker *et al*. [4]. Moreover, the activation energy calculated for temperatures between 30 and 60°C was found to be 53.2 kJ/mol, which is consistent with classic enzymatic reactions.

Additional experimental assays were performed to evaluate the quality of our model at temperatures between 40 and 60°C, at cellobiose concentrations between 0.5 and 16 mM, at glucose concentrations between 0 and 5 mM, and at enzyme concentrations between 2.5 and 10 mg/L. For each condition tested, the model predicts glucose conversion rate with high accuracy (4% error). A more important relative error was observed when very low enzyme concentrations, correlated with very low conversion level, were used.

### Comparison of β-glucosidase from *A. niger *and *T. reesei*

As detailed above, β-glucosidase from *A. niger *is generally used to complement the cellulolytic cocktail of *T. reesei*. Because of the discrepancies in the literature data, a direct enzymatic comparison between β-glucosidase from *A. niger *and *T. reesei *was performed. Using pNPG, reaction rate can be determined by measuring the amount of *p*-nitrophenol released, and high glucose concentrations can be used without affecting the accuracy of the values. The values of the kinetic parameters determined are summarized in Table 3. First order kinetic constants were higher for β-glucosidase from *T. reesei *than those from *A. niger*, corresponding to a higher hydrolysis activity. Moreover, the inhibitory effect of glucose was lower for β-glucosidase from *T. reesei*. These latter results contradict those of Grous *et al *[12], and show the necessity for comparative kinetic characterization using a purified fraction of the β-glucosidase from *T. reesei*.

**Table 3:** Comparison of β-glucosidase from *A. niger *and from *Trichoderma reesei *for the Michaelis-Menten model

	Substrate			
	
	pNPG		Cellobiose	
	
Property	*A. niger*	*T. reesei*	*A. niger*	*T. reesei*
*k cat*, per min	1582 ± 79	5276 ± 264	1897 ± 95	2445 ± 107
*K*_*m*_, mM	0.57	0.38	0.88	1.36
*K*_*p *_competitive inhibition, mM	2.70	3.25	3.40	-
*K*_*m*_/*K*_*p*_	0.21	0.12	0.26	-

pNPG, ρ-nitrophenyl-β-D-glucopyranoside.

**Figure 4:**
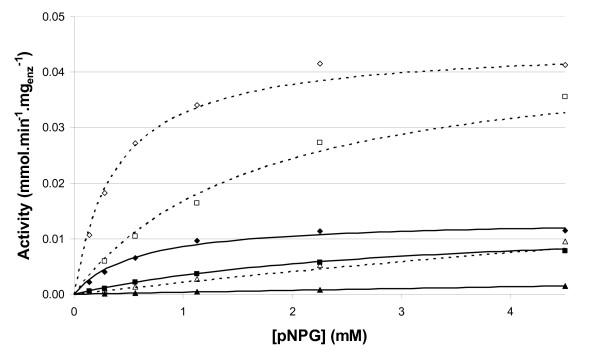
**Comparison of predicted vs. measured activities as a function of initial pNPG concentration**. Initial glucose concentration: diamond, 0 g/L, square, 2 g/L, triangle, 30 g/L. Filled symbols and solid lines, β-glucosidase from *Aspergillus niger*; empty symbols and dashed lines, β-glucosidase from *Trichoderma reesei*.

For both cellulases, inhibition parameters were determined using initial glucose concentrations up to 60 g/L. Predictions of the model versus data points determined in the presence of variable glucose concentrations are presented in Figure 4. The inhibitory effect of glucose is clearly demonstrated β-glucosidases lost 85% of their activity in the presence of 30 g/L glucose. In the process design of hydrolysis of lignocellulose, kinetic constants and influence of inhibitors are important parameters that need to be taken into account. These results confirm the necessity of characterizing each β-glucosidase and the suitability of the method used to screen different types of enzymes.

## Conclusion

This work contributes to a more reliable kinetic model of cellulolysis by the determination of kinetic parameters of purified β-glucosidase of *T. reesei *using an M-M model. In addition, this model is also suitable for the prediction of a commercial β-glucosidase preparation obtained from *A. niger *(SP188) which is frequently added into reactions to achieve complete enzymatic hydrolysis of pretreated lignocellulosic raw materials. As it integrates the complete set parameters of β-glucosidase, this model can also be used for the prediction of the action of β-glucosidase from other microorganisms. It also includes the inhibition effect of the final product (glucose) and the influence of temperature. The comparison of β-glucosidase activities on its natural substrate (cellobiose) and on a chromophoric substrate (pNPG) shows that (i) β-glucosidase affinity is higher for pNPG, (ii) the global hydrolysis activity is higher for *T. reesei *β-glucosidase and (iii) the K_m_/K_P _ratios are similar for both substrates, confirming the competitive inhibition mechanism. Thus, it validates the use of pNPG as a substrate for the determination of the kinetic parameters, which can be easily determined using the 96-well plate method described here.

Comparing the β-glucosidases of *T. reesei *and *A. niger *was previously performed by Grous *et al*. [12] but they used non-purified enzymes, which can introduce some inaccuracy in the values of parameters. Using the innovative purification protocol based on the adsorption capacity of enzymes onto cellulose, we successfully purified β-glucosidase from *T. reesei *despite its low proportion in the crude preparation. Our results show that the presence of glucose does not equally affect β-glucosidase activities from *T. reesei *or *A. niger*. Glucose inhibition can have a deleterious effect in a bioethanol production process, in which high ethanol and accordingly high sugar concentrations are expected.

As already mentioned, this β-glucosidase model is the first part of a global kinetic model of the enzymatic hydrolysis of cellulose, dissociating the activity of β-glucosidase as a final homogeneous reaction and the hydrolysis of the cellulose by endo- and exo-glucanases in a heterogeneous phase. It can be used both with supplemented and non-supplemented cocktails, and allows prediction of the cellulose saccharification

## Competing interests

The authors declare that they have no competing interests.

## Authors' contributions

MC carried out the kinetics experiments and drafted the manuscript. HM helped to develop the kinetic experiments with PCR-plates method. HM and DH purified β-glucosidase using the new purification protocol. DC reviewed and commented the manuscript. FM and NLF directed the over-all study and drafted the manuscript. All authors read and approved the final manuscript.
